# Reproducibility and responsiveness of the Symptom Severity Scale and the hand and finger function subscale of the Dutch arthritis impact measurement scales (Dutch-AIMS2-HFF) in primary care patients with wrist or hand problems

**DOI:** 10.1186/1477-7525-4-87

**Published:** 2006-11-10

**Authors:** Marinda N Spies-Dorgelo, Caroline B Terwee, Wim AB Stalman, Daniëlle AWM van der Windt

**Affiliations:** 1 Department of General Practice, VU University Medical Center, Amsterdam, The Netherlands; 2 Institute for Research in Extramural Medicine, VU University Medical Center, Amsterdam, The Netherlands; 3 Primary Care Musculoskeletal Research Centre, Keele University, Keele, UK

## Abstract

**Background:**

To determine the clinimetric properties of two questionnaires assessing symptoms (Symptom Severity Scale) and physical functioning (hand and finger function subscale of the AIMS2) in a Dutch primary care population.

**Methods:**

The first 84 participants in a 1-year follow-up study on the diagnosis and prognosis of hand and wrist problems completed the Symptom Severity Scale and the hand and finger function subscale of the Dutch-AIMS2 twice within 1 to 2 weeks. The data were used to assess test-retest reliability (ICC) and smallest detectable change (SDC, based on the standard error of measurement (SEM)). To assess responsiveness, changes in scores between baseline and the 3 month follow-up were related to an external criterion to estimate the minimal important change (MIC). We calculated the group size needed to detect the MIC beyond measurement error.

**Results:**

The ICC for the Symptom Severity Scale was 0.68 (95% CI: 0.54–0.78). The SDC was 1.00 at individual level and 0.11 at group level, both on a 5-point scale. The MIC was 0.23, exceeding the SDC at group level. The group size required to detect a MIC beyond measurement error was 19 for the Symptom Severity Scale. The ICC for the hand and finger function subscale of the Dutch-AIMS2 was 0.62 (95% CI: 0.47–0.74). The SDC was 3.80 at individual level and 0.42 at group level, both on an 11-point scale. The MIC was 0.31, which was less than the SDC at group level. The group size required to detect a MIC beyond measurement error was 150.

**Conclusion:**

In our heterogeneous primary care population the Symptom Severity Scale was found to be a suitable instrument to assess the severity of symptoms, whereas the hand and finger function subscale of the Dutch-AIMS2 was less suitable for the measurement of physical functioning in patients with hand and wrist problems.

## Background

Health status questionnaires have become increasingly popular as measurement instruments in epidemiological studies. However, the scores on these instruments can be difficult to interpret. Therefore, there is a need to define which scores or changes in scores on these questionnaires are important. We designed a 1-year follow-up study on the diagnosis and prognosis of hand and wrist complaints in primary care, in which self-administered questionnaires were used to study the impact and prognosis of hand and wrist problems. We determined the clinimetric properties of two questionnaires in a Dutch primary care population of patients with hand and wrist problems: 1) the Dutch version of the Symptom Severity Scale, assessing symptoms [1] and 2) the hand and finger function subscale of the Arthritis Impact Measurement Scales (Dutch-AIMS2-HFF), assessing physical functioning [2,3]. The two questionnaires have been found to be valid and reliable in their respective target populations: 1) people suffering from carpal tunnel syndrome and 2) people suffering from rheumatoid arthritis (RA). Our aim was to determine whether these questionnaires are also applicable in a less specific group of patients who consult their general practitioner (GP) for hand and wrist problems. We assessed the reproducibility and responsiveness of these questionnaires, and also estimated the minimal important change.

## Methods

### Questionnaires

The Symptom Severity Scale is a self-administered questionnaire that has been developed to assess the severity of symptoms in patients with carpal tunnel syndrome. This questionnaire contains eleven questions with multiple-choice responses, with a score ranging from 1 point (mildest) to 5 points (most severe) (Table 1). The total symptom severity score is calculated as the mean of the scores for the eleven individual items [1]. In a clinical study, Levine et al. demonstrated that the instrument had good reproducibility, consistency, validity and responsiveness in patients with carpal tunnel syndrome [1].

The Arthritis Impact Measurement Scales (AIMS) were designed specifically to assess health status in patients with rheumatic diseases [4]. The AIMS2 is a revised and extended version of the AIMS, and has been translated into Dutch to assess RA patients in the Netherlands [5]. The Dutch-AIMS2 is a self-administered questionnaire which measures 3 different domains of health status: physical, psychological and social aspects. In the present study we only used questions pertaining to the physical domain, namely questions about hand and finger function. The patients were asked to indicate, on a 5-point Likert scale, how often during the previous 4 weeks they had been limited in hand and finger function while performing 5 specific tasks: writing with a pen or pencil; buttoning up a shirt; turning a key; tying knots or shoelaces; opening a jar. The scores, ranging from 1 (every day) to 5 (never) for each of the items, were transformed to a total score, ranging from 0 (representing good health status) to 10 points (representing poor health status). The Dutch AIMS2 has been found to have good measurement properties [2,3,5].

### Study design and population

The study population consisted of participants in a 1-year follow-up study on the diagnosis and prognosis of hand and wrist problems. Patients were eligible for participation in the study if they were 18 years of age or older, and capable of filling in questionnaires in the Dutch language. Patients were excluded from the study if their symptoms were caused by acute trauma, injury, fracture, vascular problems or skin problems. The study was approved by the Medical Ethics Committee of the VU University Medical Centre in Amsterdam.

The first 84 participants who returned the baseline questionnaire received the Symptom Severity Scale and the Dutch-AIMS2-HFF a second time within 1 to 2 weeks after the date on which they completed the first questionnaire. These data were used to assess reproducibility. To assess test-retest reproducibility the time-interval needs to be sufficiently short to support the assumption that the condition remains stable, and sufficiently long to prevent recall [6]. The baseline and 3-month follow-up data were used to assess responsiveness.

### Data-analysis: reproducibility

Reproducibility concerns the degree to which repeated measurements in stable persons provide similar results. In other words, reproducibility is the extent to which an instrument is free of measurement error. This was assessed by rating test-retest reliability and agreement [7].

#### Test-retest reliability

As a parameter of reliability, we computed the intraclass correlation coefficient (ICC_agreement_) for the Symptom Severity Scale and the Dutch-AIMS2-HFF by using a two-way random effects model [8]. An ICC > .70 is generally considered to indicate good reliability [9].

#### Agreement

The Bland and Altman method was used to quantify agreement, by calculating the mean difference (Mean Δ) between the two measurements and the standard deviation (SD) of this difference [10]. The closer the Mean Δ is to zero and the smaller the SD of this difference, the better the agreement. The 95% limits of agreement were defined as the mean difference between the measurements ± 1.96*SD of the differences. We also computed the standard error of measurement (SEM) for both scales. The smaller the measurement error, the smaller the changes that can be detected beyond measurement error. The SEM was estimated by calculating the square root of the within subject variance of the patients (SEM = √σ_between measurement _+ σ_residual_) [7].

#### Smallest detectable change

The smallest detectable change (SDC) was based on this absolute measurement error. To be 95% confident that the observed change is real change, and not caused by measurement error, the smallest detectable change at individual level (SDC_ind_) was calculated as 1.96*√2*SEM. The smallest detectable change at group level (SDC_group_) was calculated as (1.96*√2*SEM)/√n [11,12].

### Data-analysis: responsiveness

Responsiveness refers to an instrument's ability to detect important change over time in the concept being measured [13,14]. Responsiveness can be tested by relating the smallest detectable change (SDC) to the minimal important change (MIC). The absolute measurement error should be smaller than the minimal amount of change in the scale that is considered to be important [15]. We used an anchor-based approach to determine the minimally important change for the Dutch-AIMS2-HFF and the Symptom Severity Scale. At each follow-up measurement, the patients were asked to score the change in their ability to perform daily activities. The seven response options were: (1) 'very much improved'; (2) 'much improved'; (3) 'little improved'; (4) 'no change'; (5) 'little deterioration'; (6) 'much deterioration'; (7) 'very much deterioration'. This measure of change was used as the anchor (external criterion) for the evaluation of responsiveness.

The minimal important change (MIC) was quantified by constructing receiver operating characteristic (ROC) curves [16]. The ROC curve is the result of using different cut-off points for change scores, each with a given sensitivity and specificity. To determine the MIC we defined the optimal cut-off point as the point closest to the upper left corner of the ROC curve, which is assumed to represent the lowest overall misclassification. This MIC was related to the SDC by computing the group size needed to achieve an SDC_group _that equals the MIC (n = (SDC/MIC)^2^) [11].

We also computed the area under the curve (AUC), which can be interpreted as the probability of correctly identifying an improved patient from randomly selected pairs of improved and stable patients [17,18]. An AUC of 1.0 indicates perfect discrimination between these two health states. An instrument that does not discriminate any better than chance will have an AUC of 0.50 [18].

Finally, we assessed the presence of floor and ceiling effects, by examining the frequency of the highest and lowest possible scores at baseline. Floor effects were considered to be present if more than 15% of the patients had a minimal score at baseline (1 on the Symptom Severity Scale or 0 on the Dutch-AIMS2-HFF); ceiling effects were considered to be present if 15% of the patients had a maximum baseline score (5 on the Symptom Severity Scale or 10 on the Dutch-AIMS2-HFF) [19]. The responsiveness of questionnaires is limited by the presence of floor or ceiling effects, because changes can not be measured in such cases.

All statistical analyses were performed in SPSS for Windows, Version 12.0.1.

## Results

The questionnaire was completed by 84 participants at baseline. Their mean age was 52.0 years (SD 15.6), and 74% were female. All 84 participants completed the retest Symptom Severity Scale (on average 10 days later), but 3 participants had more than 20% missing answers on the Dutch-AIMS2-HFF. These 3 cases were not included in the analysis of the Dutch-AIMS2-HFF. Table 2 presents the characteristics of the study population at baseline, including age, gender, paid job, diagnosis according to the GP, and the duration of symptoms on presentation. The three most frequent diagnoses were osteoarthritis (23.1%), Repetitive Strain Injury (RSI) (20.5%) and non-specific symptoms/unclear (20.5%). More than one quarter of the patients had suffered from their symptoms for longer than six months.

**Table 1:** The Symptom Severity Scale

1.	How severe is the hand or wrist pain that you have at night?	6.	Do you have numbness (loss of sensation) in your hand?
	1 I do not have hand or wrist pain at night		1 No
	2 Mild pain		2 I have mild numbness
	3 Moderate pain		3 I have moderate numbness
	4 Severe pain		4 I have severe numbness
	5 Very severe pain		5 I have very severe numbness
2.	How often did hand or wrist pain wake you up during a typical night in the past two weeks?	7.	Do you have weakness in your hand or wrist?
	1 Never		1 No weakness
	2 Once		2 Mild weakness
	3 Two or three times		3 Moderate weakness
	4 Four or five times		4 Severe weakness
	5 More than five times		5 Very severe weakness

3.	Do you typically have pain in your hand or wrist during the daytime?	8.	Do you have tingling sensations in your hand?
	1 I never have pain during the day		1 No tingling
	2 I have mild pain during the day		2 Mild tingling
	3 I have moderate pain during the day		3 Moderate tingling
	4 I have severe pain during the day		4 Severe tingling
	5 I have very severe pain during the day		5 Very severe tingling

4.	How often do you have hand or wrist pain during the daytime?	9.	How severe is numbness (loss of sensation) or tingling at night?
	1 Never		1 I have no numbness or tingling at night
	2 Once or twice a day		2 Mild
	3 Three to five times a day		3 Moderate
	4 More than five times a day		4 Severe
	5 The pain is constant		5 Very severe

5.	How long, on average, does an episode of pain last during the daytime?	10.	How often did hand numbness or tingling wake you up during a typical night during the past two weeks?
	1 I never get pain during the day		1 Never
	2 Less than 10 minutes		2 Once
	3 10 to 60 minutes		3 Two or three times
	4 Greater than 60 minutes		4 Four or five times
	5 The pain is constant throughout the day		5 More than five times

		11.	Do you have difficulty with the grasping and use of small objects such as keys or pens?
			1 No difficulty
			2 Mild difficulty
			3 Moderate difficulty
			4 Severe difficulty
			5 Very severe difficulty

**Table 2:** Characteristics of patients who returned the questionnaires at baseline and at 1-week follow-up

**Characteristics**	
Age in years: mean (SD) (N = 84)	52.0 (15.6)
Gender (% female) (N = 84)	74%
Paid job (N = 84)	52.4%
Diagnosis according to the GP*(N = 78)	
Rheumatoid arthritis	5.1%
Osteoarthritis	23.1%
Tenosynovitis	16.7%
Entrapment, including carpal tunnel syndrome	15.4%
Ganglion	11.5%
Repetitive Strain Injury	20.5%
Non-specific symptoms/unclear	20.5%
Other	10.3%
Duration of symptoms at baseline (N = 84)	
< 2 weeks	15.5%
3 – 4 weeks	19.0%
1 – 2 months	17.9%
3 – 6 months	21.4%
> 6 months	26.2%

* more than one answer possible; 30 patients were given >1 diagnosis

### Results concerning the Symptom Severity Scale

#### Reproducibility

The mean score at baseline, and at retest (on average 10 days later), and the mean change score are presented in Table 3. This table shows that over this period a small mean improvement was found on the Symptom Severity Scale (1–5).

**Table 3:** Test-retest reproducibility results for the Symptom Severity Scale and the Dutch-AIMS2-HFF

	N	Mean baseline (SD)	Mean 10 days (SD)	Δ Mean (SD)	Limits of agreement	ICC_agreement _(95% CI)	SEM	SDC_ind_	SDC_group_
Symptom Severity Scale* *(1–5)*	84	2.09 (0.57)	1.98 (0.69)	0.11 (0.50)	-0.87 to 1.09	0.68 (0.54 to 0.78)	0.36	1.00	0.11
Dutch-AIMS2-HFF* *(0–10)*	81	1.85 (2.09)	2.21 (2.37)	-0.32 (1.93)	-4.10 to 3.46	0.62 (0.47 to 0.74)	1.37	3.80	0.42

* higher score means worse functioning; SD = standard deviation; ICC_agreement _= intra-class correlation coefficient for agreement; CI = confidence interval; SEM = standard error of measurement; SDC_ind _= smallest detectable change at individual level; SDC_group _= smallest detectable change at group level.

Results concerning the test-retest reproducibility of the Symptom Severity Scale are also presented in Table 3. The ICC_agreement _was 0.68 (95% CI: 0.54–0.78), which indicates moderate reliability and the SDC at individual level was 20% (1.00 on a 5-point scale).

#### Responsiveness

To evaluate responsiveness we used perceived improvement in ability to perform daily activities as external criterion. The Symptom Severity Scale correlated moderately with this anchor (Spearman's rho 0.69). Table 4 shows the changes between baseline and 3-month follow-up scores for the 77 participants who completed the Symptom Severity Scale after three months. Very few patients reported a deterioration in daily functioning, and we therefore clustered the scores of patients reporting little, much or very much deterioration. The mean change scores increased with greater self-reported improvements in daily functioning.

**Table 4:** Changes in scores between baseline and 3-month follow-up for ability to perform daily activities

	**Symptom Severity Scale**				
**Daily functioning**	N	Δ ± sd	median	Percentiles	
				25th	75th

Very much improved	17	0.93 ± 0.63	1.00	0.41	1.41
Much improved	11	0.56 ± 0.39	0.45	0.27	1.00
Little improved	6	0.59 ± 0.34	0.64	0.30	0.86
No change	34	-0.03 ± 0.42	0.00	-0.14	0.18
Deterioration	9	-0.24 ± 0.38	-0.18	-0.45	0.05
	**Dutch-AIMS2-HFF**				

	N	Δ ± sd	median	Percentiles	
				25th	75th

Very much improved	16	1.47 ± 1.44	1.00	0.13	3.00
Much improved	11	2.18 ± 2.80	1.00	0.00	4.00
Little improved	6	1.10 ± 1.41	1.06	-0.13	2.25
No change	34	-0.18 ± 1.36	0.00	-0.50	0.50
Deterioration	9	-0.89 ± 2.33	0.00	-1.25	0.00

**Figure 1:**
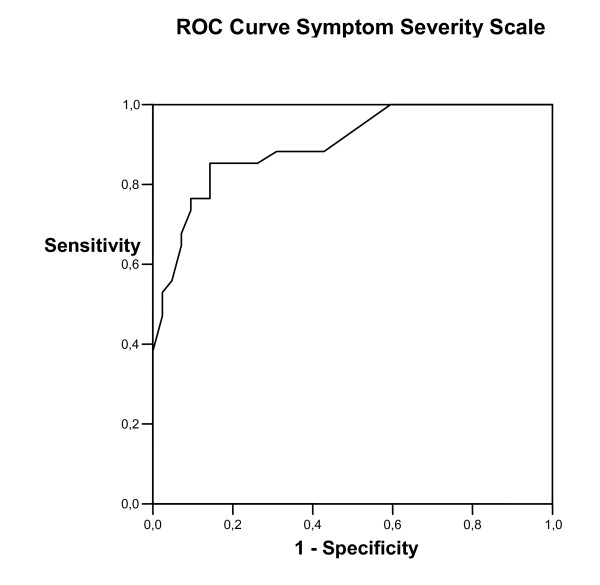
Receiver operator characteristic (ROC) curve for changes on the Symptom Severity Scale.

Figure 1 presents the ROC curves generated for changes on the Symptom Severity Scale. Based on the distribution of scores presented in Table 3, we compared patients reporting any improvement on the external criterion (n = 34) with those reporting no change (stability, n = 34). True positive rates (sensitivity) and false positive rates (1-specificity) for the discrimination between improvement and stability were plotted for multiple cut-off points. The AUC for the Symptom Severity Scale was 0.90 (95% CI: 0.83–0.97). A cut-off point of 0.23 approximates the optimal cut-off point (MIC) between sensitivity (85%) and specificity (86%).

We determined responsiveness by relating the SDC to the MIC. For the Symptom Severity Scale, the SDC_group _(0.11) was smaller than the MIC (0.23). The group size required to detect a MIC beyond measurement error was 19.

### Results concerning the Dutch-AIMS2-HFF

#### Reproducibility

Table 3 shows a small mean deterioration on the Dutch-AIMS2-HFF (0–10) between the baseline score and the retest scores. Test-retest reproducibility showed moderate reliability (ICC_agreement_: 0.62;95% CI: 0.47–0.74). The SDC_ind _was 3.80 on an 11-point scale (35%).

#### Responsiveness

The Dutch-AIMS2-HFF also correlated moderately with our anchor (Spearman's rho 0.52). Table 4 shows the mean changes for categories of improvement in daily activities in patients who completed the questionnaire after three months (n = 76). Although self-reported improvement was associated with an improvement on the scale, there was no gradual increase in scores over categories of improvement.

**Figure 2:**
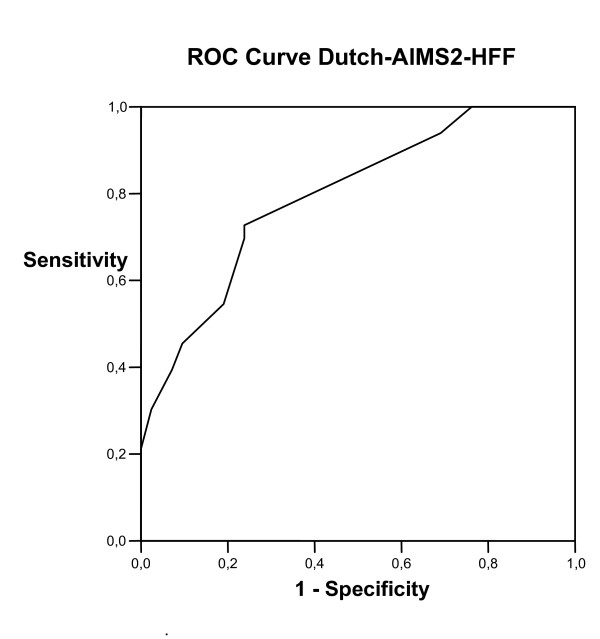
Receiver operator characteristic (ROC) curve for changes on the Dutch-AIMS2-HFF.

Figure 2 presents the ROC curves generated for changes on the Dutch-AIMS2-HFF. Again, we compared patients reporting any improvement on the external criterion (n = 33) to those reporting no change (stability, n = 34). The AUC was 0.79 (95% CI: 0.69–0.90); the optimal cut-off point (MIC) approximated 0.31 (sensitivity = 70%; specificity = 76%). The SDC_group _was not smaller than the MIC for the Dutch-AIMS2-HFF (SDC_group _of 0.42; MIC of 0.31). The group size required to detect a MIC beyond measurement error was 150. We found a floor effect for the Dutch-AIMS2-HFF; 30% of the patients had a minimum score of 0 at baseline.

## Discussion

In this study we investigated the clinimetric properties of two questionnaires, the Symptom Severity Scale and the Dutch version of the hand and finger function subscale of the Arthritis Impact Measurement Scales (Dutch-AIMS2-HFF). In our population of primary care patients with hand or wrist problems, the Symptom Severity Scale had good reproducibility and responsiveness; the Dutch-AIMS2-HFF performed less well. The measurement error (SEM) for the Symptom Severity Scale was smaller (0.36 on a 1–5 scale) than the measurement error for the Dutch-AIMS2-HFF (1.37 on a 0–10 scale). The Symptom Severity Scale detected smaller changes than the Dutch-AIMS2-HFF (20% versus 35%). The responsiveness of the Symptom Severity Scale was also better, with an AUC of 0.90, compared to 0.79 for the Dutch-AIMS2-HFF, which means that the Symptom Severity Scale discriminated better between improved and stable patients. It should be noted that we did not aim to compare the properties of the two questionnaires. Each questionnaire measures its own concept: the Symptom Severity Scale measures the severity of symptoms and the Dutch-AIMS2-HFF measures physical aspects of health status. Our aim was to examine if these questionnaires could also be applied in a group of patients for whom the questionnaires were not designed.

### Measurement properties of the Symptom Severity Scale

Levine et al.[1] tested the measurement properties of the Symptom Severity Scale in a clinical study of patients with carpal tunnel syndrome. They demonstrated that the Symptom Severity Scale is highly reproducible (Pearson's correlation coefficient, r = 0.91), internally consistent (Cronbach's alpha, 0.89), valid, and responsive to clinical change (expressed as the effect size: 1.4 for severity of symptoms).

In our more heterogeneous population, the measurement properties of the Symptom Severity Scale were found to be satisfactory.

### Measurement properties of the Dutch-AIMS2

Meenan et al. [2] tested the measurement properties of the AIMS2 in subjects with rheumatoid arthritis (RA) and subjects with osteoarthritis (OA). Internal consistency coefficients were 0.72–0.91 in the RA group and 0.74–0.96 in the OA group. Test-retest reliability was 0.78–0.94. Validity analyses in both the RA and the OA group showed that patient designation of an area as a problem or as a priority for improvement was significantly associated with a poorer AIMS2 score in that area. Meenan et al. concluded that the AIMS2 is a questionnaire with excellent measurement properties that should be useful in arthritis clinical trials and in outcome research. Riemsma et al. [3] and Evers et al. [5] assessed the reliability and validity of the Dutch version of the AIMS2 (Dutch-AIMS2). The internal consistency coefficients for the health status scales ranged from 0.66 to 0.89 [3] and from 0.65 to 0.91 [5]. Test-retest reliability with a time-interval of 1 month was high (between 0.73–0.92) [5]. The construct validity of the Dutch-AIMS2 was confirmed by the results of factor analysis, which identified the three different domains [3,5].

In our study the MIC for the Dutch-AIMS2-HFF was small (0.31), but the measurement error was so large that the MIC could not be discriminated from measurement error. The Dutch-AIMS2-HFF was developed for the assessment of patients with RA [2,3], whereas the patients in our study suffered from a variety of hand and wrist problems. It is possible that the Dutch-AIMS2-HFF is not suitable for this more heterogeneous primary care population. The presence of a floor effect seems to confirm this suggestion; because many patients (30%) reported no limitation in hand and finger function (score 0) at baseline, it was not possible to detect any improvement in these patients.

Another possible explanation for the poorer performance of the Dutch-AIMS2-HFF may be the number and nature of its items. It contains only five questions, all of which concern almost equally difficult functions. This may affect the ability of the instrument to measure within-subject change.

### Methodological considerations

The baseline results showed that almost 50% of the patients had suffered from their symptoms for more than three months, and could therefore be defined as chronic. It is plausible to assume that test-retest reliability would be higher in patients with chronic symptoms than in patients with acute or sub-acute symptoms. We performed a sub-group analysis, in which we compared the ICC_agreement _between patients with chronic symptoms to that of patients with more acute symptoms. The results showed very small differences, indicating that the duration of symptoms did not affect test-retest reliability.

In our study we used the scores for perceived change in ability to perform daily activities as external criterion (anchor) for assessing responsiveness. We could, however, have opted for pain improvement, or scores for overall improvement, but these other options did not correlate any better with the two questionnaires than the external criterion that we used. A correlation of more than 0.5 is considered to be appropriate when selecting an external criterion for assessing responsiveness [20].

We used an anchor-based approach to determine the MIC. However, there are also several other methods that can be used to determine MIC; for example, Jaescke et al. [21], Norman [22] and Wyrwich [23] used other methods. Jaescke et al. used the mean change score in people reporting a small improvement to determe the MIC. With this method, the MIC for the Symptom Severity Scale would be 0.59 (the mean change among patients reporting little improvement), and for the Dutch-AIMS2-HFF it would be 1.10. Norman et al. found that under many circumstances the estimates of MIC fall very close to half a SD_baseline_. With this method, the MIC for the Symptom Severity Scale would be 0.29 and for the Dutch-AIMS2-HFF it would be 1.05. Wyrwich proposed one SEM as a measure for MIC [24]. Following this method, the MIC for the Symptom Severity Scale would be 0.36 and for the Dutch-AIMS2-HFF it would be 1.37. The anchor-based approach we used estimates the change score at which the questionnaires discriminate best between improved and stable patients. This method results in smaller MIC estimates, compared to the other methods, but may be closer to the *minimal *important change. The definition of an optimal cut-off point (MIC) may depend on the objective for which the questionnaire is used. For example, if users (researchers or clinicians) want to be certain that *only *improved patients are identified by the questionnaire, a higher cut-off score can be defined for the MIC, but this approach will fail to identify more patients with smaller, yet important changes. We prefer to use the ROC curves for defining MIC, because this method clearly illustrates the consequences of selecting different MICs.

## Conclusion

In conclusion, the properties of a questionnaire always depend on the characteristics of the population in which the questionnaire is used. In our heterogeneous, primary care population, the Symptom Severity Scale seems to be a suitable instrument to assess the severity of symptoms, whereas the hand and finger function subscale of the Dutch-AIMS2 seems to be less suitable for the measurement of physical functioning in patients with hand and wrist problems.

## Competing interests

The authors declare that they have no competing interests.

## Authors' contributions

DvdW and MS developed the protocol and DvdW secured the funding. MS was responsible for the organization, collected the data, carried out the statistical analyses together with DvdW and CT, and wrote the original draft. DvdW, CT and WS contributed substantially to the interpretation of the data, revised the draft critically with regard to important intellectual content, and approved the final version of the paper.

## Appendix

The Symptom Severity Scale [1].
